# ‘Tidy’ and ‘messy’ management alters natural enemy communities and pest control in urban agroecosystems

**DOI:** 10.1371/journal.pone.0274122

**Published:** 2022-09-22

**Authors:** Monika Egerer, Stacy M. Philpott

**Affiliations:** 1 Environmental Studies Department, University of California—Santa Cruz, Santa Cruz, CA, United States of America; 2 Department of Life Science Systems, TUM School of Life Sciences, Technical University of Munich, Freising, Germany; University of Manitoba, CANADA

## Abstract

Agroecosystem management influences ecological interactions that underpin ecosystem services. In human-centered systems, people’s values and preferences influence management decisions. For example, aesthetic preferences for ‘tidy’ agroecosystems may remove vegetation complexity with potential negative impacts on beneficial associated biodiversity and ecosystem function. This may produce trade-offs in aesthetic- versus production-based management for ecosystem service provision. Yet, it is unclear how such preferences influence the ecology of small-scale urban agroecosystems, where aesthetic preferences for ‘tidiness’ are prominent among some gardener demographics. We used urban community gardens as a model system to experimentally test how aesthetic preferences for a ‘tidy garden’ versus a ‘messy garden’ influence insect pests, natural enemies, and pest control services. We manipulated gardens by mimicking a popular ‘tidy’ management practice–woodchip mulching–on the one hand, and simulating ‘messy’ gardens by adding ‘weedy’ plants to pathways on the other hand. Then, we measured for differences in natural enemy biodiversity (abundance, richness, community composition), and sentinel pest removal as a result of the tidy/messy manipulation. In addition, we measured vegetation and ground cover features of the garden system as measures of practices already in place. The tidy/messy manipulation did not significantly alter natural enemy or herbivore abundance within garden plots. The manipulation did, however, produce different compositions of natural enemy communities before and after the manipulation. Furthermore, the manipulation did affect short term gains and losses in predation services: the messy manipulation immediately lowered aphid pest removal compared to the tidy manipulation, while mulch already present in the system lowered Lepidoptera egg removal. Aesthetic preferences for ‘tidy’ green spaces often dominate urban landscapes. Yet, in urban food production systems, such aesthetic values and management preferences may create a fundamental tension in the provision of ecosystem services that support sustainable urban agriculture. Though human preferences may be hard to change, we suggest that gardeners allow some ‘messiness’ in their garden plots as a “lazy gardener” approach may promote particular natural enemy assemblages and may have no downsides to natural predation services.

## Introduction

In agroecosystems, ecological complexity often supports higher biodiversity and ecosystem function. Greater vegetation diversity and structure, for example, greater cultivated and wild crop diversity, woody vegetation, and floral abundance and diversity, can enhance pest control services by providing habitat and resources for natural enemies that regulate pests [1–4]. Furthermore, farmers that allow ‘weeds’ to grow are adding a dimension of this vegetation complexity that may contribute to habitat heterogeneity and natural enemy habitat provisioning to enhance antagonistic interactions between agricultural pests and predators [5, 6]. Thus, principles underlying agroecology maintain that adding ecological complexity to systems through practices such as vegetation diversity, complexity and connectivity, and through ground cover structural complexity, should theoretically support the biodiversity of ecosystem service providers and enhance ecosystem service provision.

In social systems, however, ecological complexity may not synergize with human aesthetic preferences that influence ecosystem management [7]. As portrayed in 17^th^ and 18^th^ century art of the Judeo-Christian tradition, some have historically preferred idyllic natural landscapes composed of symmetry and order, not of ecological complexity [8, 9]. Work in environmental psychology shows that people may favor “tidy” and “manicured” nature scenes rather than “messy” or “unorderly” nature scenes [10], a preference that humans have evolved [11], though such relationships often depend on socio-cultural context [12]. In contemporary Western culture, tidy landscapes, including mowed turfgrass lawns, and ‘weed’ free agricultural fields and urban gardens are an expression of domination and control, social capital and norms [13, 14], and ‘cues of care’ [7]. Such preferences have transformed urban landscapes into low-diversity urban forests and turfgrass lawns [13, 15–17]. Under the principle that cultivation improves urban nature, unattractive vegetation often does not survive under the mantra ‘first cleaning, then greening’ in cities [18, 19]. Thus, ‘*tidy*’ systems with orderly and structured vegetation and ground cover features are often preferred over ‘*messy*’ systems with more vegetation cover and structure [7], even if the latter can promote ecological complexity and ecosystem function [8, 20].

This apparent tension and conflict between ecosystem function on the one hand and human preference and management behavior on the other is an area ripe for research, especially within urban environments [21, 22]. ‘Messy’ ecosystems may engender ecosystem service benefits, but messiness may produce trade-offs: the habitat heterogeneity and vegetation availability that ‘messy’ looking plant species provide to natural enemies can be at odds with the aesthetic preferences and perceptions of urban residents. For example, in urban pocket prairies, high vegetation structural complexity promoted pollinators, but elicited feelings of unsafety and disdain by neighborhood residents [23]. In other examples, converting lawns into tall-grass meadows has been shown to enhance biodiversity, but this impacted people’s sense of usability for outdoor activity such as recreation [20, 21, 24]. Thus, ecological strategies for regulating services like pest control are not necessarily synergistic with land managers, ecological practices and associated communities (even if urban residents may have strong environmental beliefs) and cultural services around green space use [25, 26].

Urban agroecosystems–e.g., urban allotment and community gardens designed for food and flower production–provide a model system to investigate the coalescence between ecological complexity, aesthetic preferences, and management, in providing for biodiversity and ecosystem service provision. These types of agroecosystems are biodiverse ecosystems in urban regions designed around crop production, and heavily managed by a range of stakeholders. Limited ecological research in urban gardens suggests that urban agroecosystem ‘messiness’–allowing spontaneous vegetation and dead plant matter, and use of less mulch or woodchip ground cover–can have surprising biodiversity benefits for the abundance and diversity of organisms (bees, beetles, birds, parasitoid wasps) and ecosystem functions (pest regulation, pollination). Vegetation diversity influences natural enemy taxonomic diversity and community composition in urban agroecosystems [27], which has inspired research that investigates pest control services in relation to these measures, often with limited and unclear results [28]. While greater urban agroecosystem crop diversity may influence the assemblage of natural enemies and community similarity within the system, we do not know how this relates to pest control. Other work that compares vacant lots with high weedy vegetation to urban agroecosystems (allotment gardens) suggests that high complexity through weedy vegetation volume with low maintenance could boost natural pest control due to increased vegetation connectivity and thereby natural enemy movement and activity [29]. In urban gardens in California, ladybird beetle richness is higher in gardens with less woodchip mulch and more trees and shrubs [30], while the composition of parasitoid wasp communities is driven by the herbaceous plant cover and mulch cover in gardens [31]. In other areas, mulch may negatively affect ant abundance [32] or boost spider numbers [33] whereas plant diversity may benefit ladybeetles [34]. Thus, management features may have differential effects depending on location and taxon examined. Here, communities may be more similar when there is greater vegetation connectivity in the garden landscape, compared to gardens where vegetative connectivity is disrupted with e.g., woodchip mulch. Pest predation is lower in these gardens with more woodchip mulch, but higher in gardens with more leaf litter, more trees and shrubs, and with more and taller vegetation [35]. Collectively, this evidence suggests that management characteristics that represent ‘tidiness’ (e.g., employing woodchip mulch) versus ‘messiness’ (e.g., having generally more vegetation and ground cover heterogeneity) may produce different biodiversity outcomes around species richness and community composition, and ecosystem function outcomes relevant to sustainable urban crop production.

In this study, we investigate how the biodiversity of natural enemies and pest predation services are affected by urban agroecological management practices driven by aesthetic preferences of gardeners. Specifically, we examined how increasing ‘tidiness’ and increasing ‘messiness’ in gardens affects herbivores, arthropod natural enemies, and pest removal. Through an experimental tidy/messy manipulation of gardens, we asked: Do changes in garden ‘tidiness’ or ‘messiness’ lead to a change in herbivore densities, natural enemy communities, and pest predation services in gardens? We hypothesized that increasing ‘tidiness’ (i.e., decreasing vegetation complexity, plant structure, and connectivity) and thereby decreasing ‘messiness’ would *(H1)* decrease natural enemy abundance, richness and predation services, and *(H2)* decrease natural enemy community similarity (abundance of different natural enemy groups) in garden beds within the treatment area where beds are isolated from one another. In ‘tidy’ areas with less vegetation and more mulch in pathways, garden beds may appear functionally isolated to arthropods who may preferentially forage on vegetation, or who use vegetation as shelter from predators while foraging on the ground; on the other hand, in ‘messy’ areas with increasing vegetation complexity and structure, garden beds may be more functionally connected to one another to increase arthropod community similarity.

## Materials and methods

### Study system

We used eight urban community gardens for our experiment. We chose these eight gardens from >30 gardens where we have done previous urban agroecological research on herbivore-natural enemy interactions and predation services (see e.g., [35–37]). We were limited to the eight specific gardens because (1) we only wanted to include allotment gardens where individuals or families manage distinct garden plots or beds, and (2) we received management and gardener permission to do a manipulative experiment only in only eight of the allotment gardens in our garden network. The eight gardens in this study vary in size (445–6,070 m^2^), age (4–37 years in cultivation), habitat management, and urban landscape context from one another [35, 38]. The gardens are used for food production and ornamental flower production, which creates a wide diversity of plants grown [39]. All of the gardens are located within the Monterey Bay Plains Ecoregion characterized by a Mediterranean climate regime. The difficulty in replicating the experimental manipulation across the region (site availability, time) limited our ability to increase garden treatment replication numbers and we recognize as a limitation in our study.

The experiment was conducted from June 15 to 29, 2019, and divided into three time points in which we performed the experiments and collected data: *(i)* before the tidy/messy manipulation (“t_0_”); *(ii)* three days immediately after the tidy/messy manipulation (“t_1_”); and *(iii)* seven days after the tidy/messy manipulation (“t_2_”).

### Manipulating “tidy” and “messy” aesthetic practices

**Fig 1:**
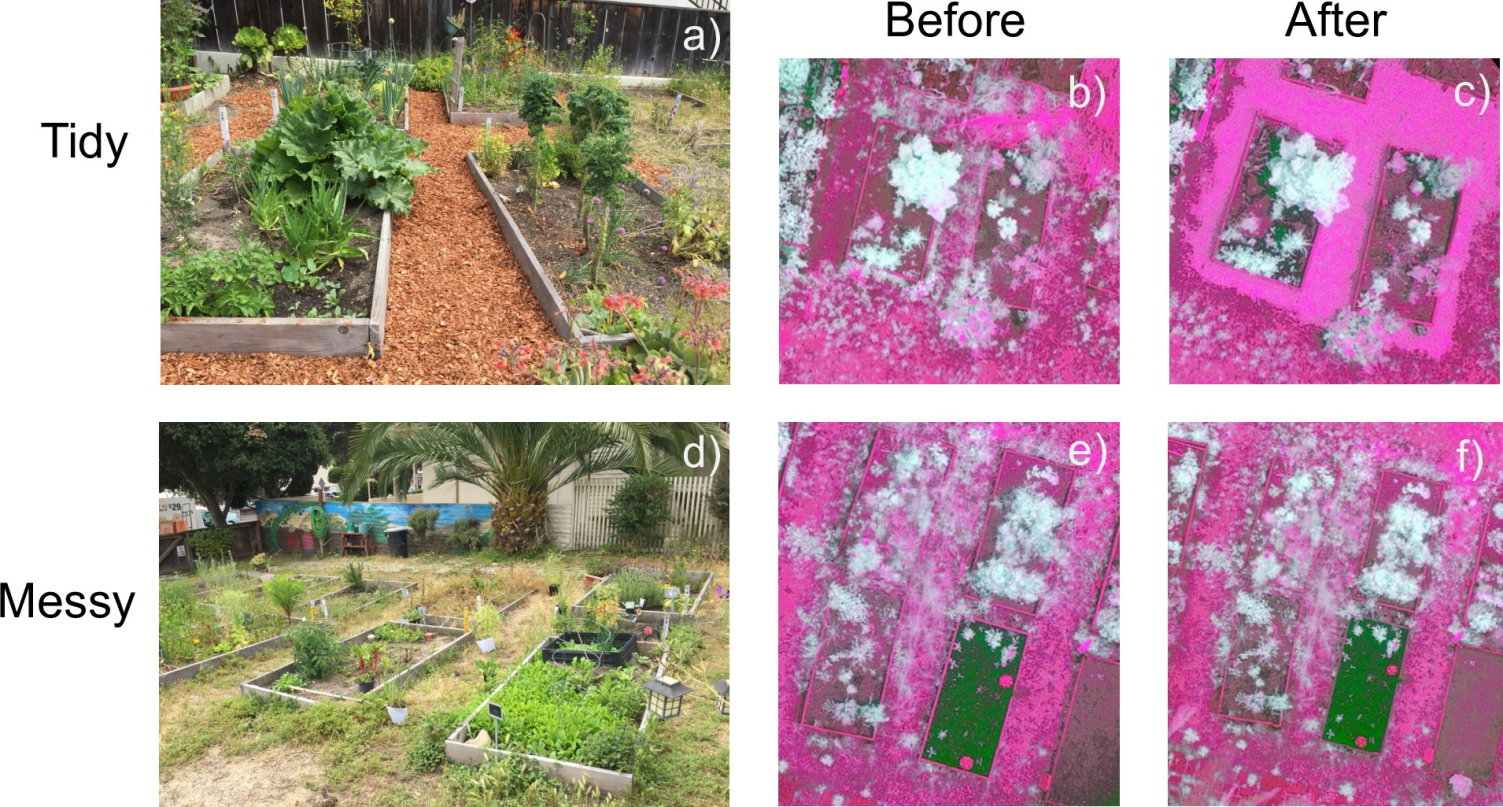
We implemented a ‘tidy’ and ‘messy’ manipulation in eight urban community gardens in the California Central coast. Example of the ‘tidy’ manipulation (a), with images taken before (b) and after (c) the manipulation; example of the ‘messy’ manipulation (d), with images taken before (e) and after (f) the manipulation. Images b, c, e, f are images taken from a compact commercial multirotor unmanned aerial vehicle (UAV) used to carry out autopiloted flights over the gardens for an aerial view of the manipulation. For panels b, c, e, and f, white areas are vegetation, green areas are bare soil, and pink areas are impermeable material such as wood, mulch, thatch, or straw.

Within each of the eight gardens, we established two spatially separate treatment areas to manipulate. Each treatment area consisted of four contiguous garden beds (i.e., garden boxes), blocked together into a ‘tidy’ treatment area and a ‘messy’ treatment area (Fig 1A, 1D and S1 Fig). We selected these specific garden beds and treatment areas based on permission from gardeners. Garden beds were on average 5.5 m^2^ in size, with each bed separated on average by 1.5 m pathways. We applied the ‘tidy’ and ‘messy’ treatment to the four garden beds, plus all surrounding pathways, thus each treatment area was on average 85 m^2^. Within a garden, the ‘tidy’ and ‘messy’ areas were separated from each other by between 5–10 m. The manipulations were designed to represent common management practices observed in gardens that represent people’s aesthetic preferences for their gardens. For our ‘tidy’ manipulation, we manipulated the ground cover within pathways using store-bought redwood mulch woodchips, a common ground cover amendment used to suppress weedy vegetation. In implementing this treatment, we did not aim to separate out the various mechanisms by which mulch may affect arthropods and their interactions (e.g., off-gassing and interrupting chemical communication, increasing soil organic matter through decomposition, weed suppression, etc.). Rather, we aimed to focus on the overall community-level effect of this ‘tidy’ manipulation. In addition, we removed all spontaneous vegetation (i.e., ‘weeds’) within the pathway area around the four garden beds. For our ‘messy’ manipulation, we manipulated the vegetation availability within the pathways of the gardens also under the hypothesis that adding more weedy vegetation (biomass, structural complexity) in addition to what was there could further promote more connectivity of natural enemies and thereby influence community assembly and pest removal. We placed potted plants of three common plant species in our system (*Bromus carinatus* (California Brome Grass), *Conyza canadensis* (Erigeron or Horseweed), and *Vicia villosa* (Vetch)) within the pathways at a density of one plant per m^2^ for about 10–12 plants per garden. We estimate that adding these potted plants added approximately ~7.3% percent vegetation cover on average to the plots, with an average vegetation cover within the ‘messy’ treatment areas of 65.7% (ranging from 42–89%). We chose these plant species for their aesthetic characteristics as ‘weedy’ plants with crowns of high width and surface area. All plants were grown at the Thimann Greenhouse at the University of California, Santa Cruz under standard conditions. Plants were watered and maintained throughout the experiment.

### Sampling natural enemies and herbivores

We sampled for herbivores and natural enemies within tidy and messy manipulation areas using visual surveys for herbivores and natural enemies, and pitfall traps for natural enemies.

For the visual surveys, at each time point, we carefully placed two edges of a 0.25 m x 0.25 m quadrat within the center of each of four garden beds in each tidy/messy area and counted and collected (or identified) natural enemies and herbivores seen on the ground or on vegetation within the quadrat in 5 min, gently searching plant matter and ground cover. We did not leave time between placing our quadrats and surveys, but placed quadrats as carefully as possible so as to not disturb the vegetation. We targeted our natural enemy surveys on predators including, but not limited to, spiders (Class: Arachnida) and lady beetles (Family: Coccinellidae) as well as groups of omnivorous insects that can be important predators in agroecosystems such as ants (Family: Formicidae), ground beetles (Family: Carabidae), earwigs (Family: Forficulidae), and harvestmen (Order: Opiliones). We targeted our herbivore surveys on aphids (Family: Aphididae) and lepidopteran larvae (Order: Lepidoptera) but noted if other groups were seen. Surveys were done by the same two observers throughout the experiment in minimum weather conditions of partly cloudy and >16°C from ~09:00–18:00.

We placed one pitfall trap in each garden bed in each tidy/messy area at each time point for 24 h. The pitfall traps indicate activity density, not necessarily abundance of natural enemies. We used 12 oz. clear plastic tubs (11.4 cm diameter × 7.6 cm deep) for pitfall traps. We placed one trap at the center of each garden bed, buried flush to the soil level and filled traps with 200 mL of a saturated saline solution with a drop of unscented detergent to break the surface tension. Upon collection of pitfall traps, we rinsed arthropods with water, separated them to order, and then stored insects in vials with 70% ethanol.

We identified herbivores to order and morphospecies level and in the case of aphids to family level. We identified natural enemies to order and further identified spiders and ground beetles to family and lady beetles and ants to species. We used the identification to order, however, for our species richness analysis and community composition analysis (see ‘Data Analysis’ below) to avoid singletons (particularly for the community composition analysis) and to maintain consistency across trophic levels analyzed and to maximize the number of sampling points. We combined pitfall trap data and visual surveys for each time point to maximize our sampling effort to get an overall assessment of the natural enemies within the short time frame.

### Sampling predation services

To determine whether the tidy/messy manipulation affected predation services provided by naturally occurring predator species in gardens, we conducted sentinel pest removal experiments with two types of prey: *(i)* corn earworm eggs (*Helicoverpa zea*) and *(ii)* pea aphids (*Acyrthosiphon pisum*). We only focused on the removal of prey, and not parasitism. We purchased eggs from Frontier Agricultural Sciences in Newark, DE, USA and aphids from Berkshire Biological in Westhampton, MA, USA. All insects were purchased and transported under USDA-Aphis permit P526P-14-02660, and all insects were destroyed after the experiment. Eggs were laid on cloth sheets, which we cut into 1 cm × 1 cm squares (mean 600 eggs per square), and stored in a freezer for at least one week prior to field experiments. Prior to the field experiment, we marked treatment and site for all egg squares, and photographed egg squares under a microscope. After the field experiment, we returned egg squares to the lab, photographed them again, and then from photos counted the number of eggs before and after field placement. For field experiments, we pinned egg squares onto two leaves (one open to natural enemies and one bagged to exclude natural enemies) of potted, greenhouse-raised 20 cm tall *Brassica* plants. We then placed two sets of *Brassica* plants in each garden, one set (one open plant, one bagged plant) in the ‘tidy’ area and one set in the ‘messy’ area. Plants were placed next to each other in the pathway neighboring the edge of beds in each area in each garden, and we collected all egg squares after 24 hr. Because eggs were frozen for a week, we assume that all missing eggs were due to predation (open plant) or perhaps falling off of the sheets (open and bagged plants), and not due to eclosed eggs. Aphids were reared on covered fava bean (*Vicia faba*) plants in the Thimann Greenhouse at UC Santa Cruz until populations reached ~600–1200 aphids per plant. As with eggs, for field experiments we placed two plants, one open plant and one bagged plant (no natural enemy access) in pathway locations in each tidy/messy area, with open and bagged plants placed in the same spot. Ground dwelling predators had access to the sentinel eggs and aphids. We counted the number of aphids on plants upon arrival at the garden, and had the same person count the number of aphids on the same plants before and after the experimental period to reduce observer bias. We returned 24 h later to retrieve plants, and recounted the aphids on the plants before leaving the garden. For both prey items, we noted whether any predators were present inside bags (i.e., enclosure treatments were ineffective).

### Measuring bed- and garden-scale management

To control for and explore impacts of different local scale garden management practices in the gardens, we measured vegetation and groundcover management at each time period at two spatial scales in the gardens: *(i)* within each of the four individual garden beds within each tidy/messy manipulation area in each garden; and *(ii)* within 20 m x 20 m sampling plots placed at the center of each garden. The tidy and messy treatment areas were also located within the 20 m x 20 m sampling plots.

Within each of the eight garden beds (four in each tidy/messy area) at each site, we identified all herbaceous plants (except grasses) to morphospecies, counted all flowers (including inflorescences), and assessed the percent ground cover composition within the bed. For each bed, we calculated a vegetation complexity index (VCI) of the herbaceous plant variables including measurements of herbaceous vegetation cover, vegetation height, number of flowers, number of flower species, and the number of herbaceous species. For the VCI, we scaled all values for these variables to a scale from 0 to 1 (with 1 being the highest value measured across all sites and 0 the lowest value measured across all sites) so that each variable was given equal weight in the index, and so that all values were comparable across all gardens to one another. Then we averaged scaled variable values to create the VCI. Thus, this index for each garden represents the averaged value taken across all measures, scaled relative to the highest value within each variable.

At the garden scale (20 m x 20 m plot), we collected information on local vegetation and ground cover by randomly placing eight 1 m x 1 m quadrats within which we identified all herbaceous plants (except grasses) to morphospecies, counted all flowers, and assessed percent ground cover composition (herbaceous vegetation, grass, bare ground, mulch, and rocks). We similarly calculated a VCI of the herbaceous plant variables at this scale.

### Data analysis

We performed three statistical analyses to determine whether our manipulation of garden ‘tidiness’ or ‘messiness’ changed the abundance of herbivores, abundance and species richness of natural enemies, and predation services throughout the experiment: *(i)* non-parametric Mann-Whitney-Wilcoxon tests comparing treatment means for changes in response variables between each time point; *(ii)* generalized linear models (GLMs) examining the extent to which local management practices predicted post-experiment response variables; and *(iii)* constrained multivariate analysis (redundancy analysis) and post hoc pairwise tests to examine the change in the community composition of natural enemies.

#### Testing for significant changes between time points, between tidy and messy

For all response variables, we calculated the difference in values from before the manipulation to both time points after the manipulation (t_1_-t_0_; t_2_-t_0_), resulting in two change values for each response variable for each site. The response variables included change in: *(i)* natural enemy activity and richness (pitfall traps and visual surveys), *(ii)* herbivore abundance (visual surveys), *(iii)* and predation services (experimental plants). Predation services were measured as predation effect sizes (log response ratio, LRR), calculated as *ln(proportion prey removed in open treatments) − ln(proportion of prey removed in bagged treatments)* for each site, for each treatment. Higher values indicate higher removal of prey items from open relative to bagged plants. We then used Mann-Whitney-Wilcoxon Test to compare whether the tidy/messy manipulation (‘messy’ vs ‘tidy’; predictor variables) influenced the change in response variables between before and both post-manipulation time points. We used this approach to specifically test whether the tidy/messy manipulation influenced a change in biodiversity of natural enemies or herbivores or predation services.

#### Hypothesis testing to predict biodiversity and predation services

Second, we built generalized linear models (GLMs) to predict herbivore and natural enemy abundance/activity and richness (order level), and predation services across both time periods. For each response variable (n = 6), we built models with manipulation (tidy vs messy) and garden management factors (mulch cover within garden beds, mulch cover in the 20 m x 20 m plot, VCI of herbaceous plants within garden beds, and VCI of herbaceous plants in the 20 m x 20 m plot) as covariates (n = 30 models). Predation service models were fit with a Gaussian distribution; natural enemy abundance and natural enemy richness models were fit with a Poisson distribution for count data; herbivore abundance was fit with negative binomial distribution. VCI and mulch values were scaled in the models, and VIF scores for all models were <2. Significance of the explanatory variables was taken at P ≤ 0.05. Model fit for each model was estimated using Akaike information criterion for small sample sizes (AICc) relative to a null model with none of the explanatory variables [40].

#### Testing for post-manipulation natural enemy community shifts in tidy and messy treatments

To determine whether the tidy/messy manipulation affected the community composition of natural enemies, we utilized constrained multivariate analysis–redundancy analysis (RDA)–to measure how much the variation in the composition of natural enemy communities is explained by the tidy/messy manipulation. We used a constrained method because of our *a priori* hypotheses about the factors that affect composition (i.e., tidy versus messy manipulation). For each time point, we created a matrix of the variation in total order composition, removed singletons, and applied a Hellinger transformation using *vegan* [41] in R to standardize abundance across orders. We used analysis of variance to evaluate the statistical significance of the constraint. To determine whether there were significant differences in natural enemy community composition in orders before and after the tidy/messy manipulation, we used Procrustes analysis using the ‘‘protest” function in *vegan* to assess similarity among ordinations. To determine whether there were significant differences in natural enemy community composition between tidy/messy manipulation areas, we performed an analysis of similarity test (ANOSIM) using the ‘‘anosim” function in vegan between treatments for each time point. The ANOSIM test compares the mean distance within a group to the mean distance between groups to statistically determine the difference in species composition between the two different treatments. We then performed a PERMANOVA using the “adonis2” function in vegan for each time point to compare within treatment mean distance between points to a centroid. In addition, we performed a Procrustes test (i.e., a type of multidimensional scaling that tests the similarity of two data sets, such as species distribution, by analyzing the distribution of sets of shapes in relation to one another; [42]) between each time point (three tests: t_0_-t_1_; t_1_-t_2_; t_0_-t_2_). This method allowed us to match corresponding sampling points (here, each garden bed within a treatment within each site) from each of the data sets. Finally, we conducted a SIMPER analysis using the “simper” function in vegan for each time point (as above) to determine which natural enemy groups were responsible for differences within and between treatments. This analysis tests pairwise comparisons of treatments to determine the average contribution of each taxonomic group to the average overall Bray–Curtis dissimilarity [43].

Analyses were all performed in the R statistical environment [44].

## Results

We documented a diversity of natural enemies and herbivores throughout the gardens over the course of the experiment (Table 1) and some changes in predation services from the tidy/messy manipulation.

**Table 1:** Summary of the numbers (counts) of natural enemies observed in pitfall traps (a) and visual surveys (b), and the summary of herbivores observed in visual surveys. Counts of orders and families include counts of genera and species.

	Order	Count (all)
**Natural enemies**		
a) Pitfall	Araneae	130
	Coleoptera	72
	Dermaptera	17
	Hymenoptera	262
	Opiliones	63
	Unknown	6
	Total	550
b) Visual	Araneae	123
	Coleoptera	61
	Hymenoptera	497
	Opiliones	19
	Total	700
	** **	**1250**
**Herbivores**		
	Coleoptera	22
	Hemiptera	66
	Lepidoptera	33

### Effects on herbivores and natural enemies

**Fig 2:**
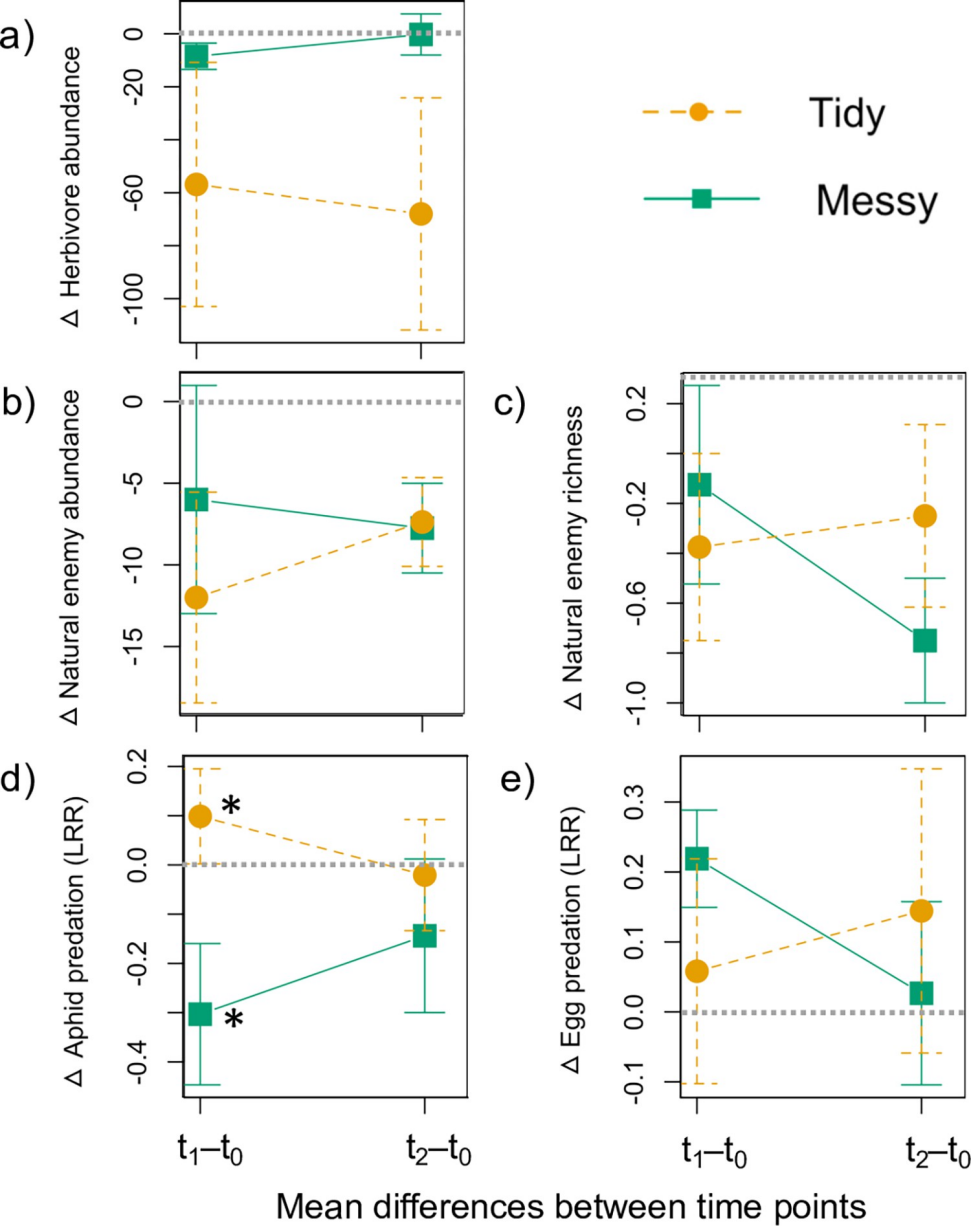
Trends in the changes (Δ) in herbivores (a), natural enemies (b, c), and pest predation (d, e) between the first and second observations. Graphs show raw value means across the 16 beds within each treatment area in each of the eight gardens. Positive values indicate a higher response variable value after the experiment, whereas negative values indicate a lower response variable value. Gray dashed horizontal lines indicate no change between time points. Error bars represent standard errors (SE) of the means of all data points (garden bed observations). Time points include before experimental manipulation (t_0_), three days after the manipulation (t_1_) and seven days after the manipulation (t_2_). Asterisks (*) indicate significant differences between treatments at a respective time point tested using Mann-Whitney-Wilcoxon Tests (Significance: ‘*’ 0.05, ‘**’ 0.01, ‘***’ 0.001).

Herbivore and natural enemy abundance and richness were not strongly impacted by the tidy/messy manipulation. The abundance of herbivores observed was similar in the messy treatment between time points and similar between time points in the tidy treatment (Fig 2). Herbivore abundance was not influenced by any factors in the GLMs (Table 2).

**Table 2:** Generalized linear models built to predict herbivore, natural enemy, and predation services. Here we present the best models for each response variable (models <2 AIC in difference included in the table for each response variable). Abundance and richness for herbivores are from visual surveys. Abundance and richness for natural enemies are combined values from visual surveys and pitfall traps. Intercepts are scaled values. Significance is denoted as (*) for P values, as * between 0.01–0.05, as ** for values <0.01, as *** for values <0.001. (AIC = Akaike Information Criterion; df = degrees of freedom; LRR = Log Response Ratio; VCI = vegetation complexity index).

Model (per response)	Response factor	Predictor factor	Estimate	Std. Error	df	T-value	Pr(>|t|)	AIC	Δ AIC
**1a**	Herbivore abundance	Intercept	2.13	0.30	31	7.18	<0.001	192.67	0.00
**1b**	Herbivore abundance	Intercept	2.12	0.30	30	7.18	<0.001	194.55	1.88
		Pre-manipulation abundance	0.11	0.30	30	0.37	0.71		
**1c**	Herbivore abundance	Intercept	2.11	0.42	30	5.03	<0.001	194.66	1.99
		Tidy/messy manipulation	0.04	0.59		0.06	0.95		
**2**	Natural enemy abundance/activity	Intercept	3.12	0.04	30	83.26	<0.001	320.55	-35.71
		Pre-manipulation abundance	0.22	0.04		6.22	<0.001		
**3**	Natural enemy order richness	Intercept	1.13	0.10	30	11.14	<0.001	106.33	-0.30
		Pre-manipulation richness	0.16	0.11		1.50	0.135		
**4a**	Aphid predation (LRR)	Intercept	1.30	0.05	31	27.37	<0.001	9.78	0.00
**4b**	Aphid predation (LRR)	Intercept	1.30	0.05	30	27.37	<0.001***	10.73	0.96
		Pre-manipulation LRR	0.05	0.05		1.00	0.33		
**4c**	Aphid predation (LRR)	Intercept	1.26	0.07	30	18.65	<0.001***	10.80	1.03
		Tidy/messy manipulation	0.09	0.10		0.96	0.34		
**5**	Egg predation (LRR)	Intercept	1.19	0.08	28	15.36	<0.001***	21.64	-2.85
		Tidy/messy manipulation	0.02	0.11		0.14	0.89		
		VCI	-0.09	0.06		-1.61	0.12		
		Mulch in garden	-0.14	0.06		-2.54	0.017*		

Changes in natural enemy abundance and richness between treatment areas (i.e., all beds combined) did not differ between time points (Fig 2). The tidy/messy manipulation did not strongly impact post-manipulation total natural enemy abundance/activity or richness between the treatments. The best models predicting abundance/activity and order richness only included pre-manipulation abundance/activity and richness values (Table 2). The ANOSIM test (comparison within and among treatment distance between data points) found no difference in the messy/tidy composition before the manipulation (R = -0.000; P = 0.419) or after the manipulation, regardless of time point (t_1_: R = -0.019; P = 0.779; t_2_: R = -0.018; P = 0.778). Furthermore, the PERMANOVA test revealed that there were no strong differences in the spread of points between the treatments before the manipulation (F = 1.368; P = 0.255) or after the manipulation at either time point, t_1_ (F = 0.446, P = 0.713) or t_2_ (F = 0.402, P = 0.706). The SIMPER test found that Hymenoptera (30.0%), Coleoptera (18.1%) and Araneae (11.8%) abundance contributed the highest amount to average between-group dissimilarity before the manipulation. The patterns were similar for after the manipulation at both time points: t_1_ (Hymenoptera = 28.3%; Coleoptera, 13.2%; Aranea, 13.0%) and t_2_ (Hymenoptera = 28.3%; Coleoptera, 15.9%; Araneae, 15.4%). However, across all time points, the probability of getting a larger or equal average contribution of these orders to a treatment was not significant (p>0.10).

**Fig 3:**
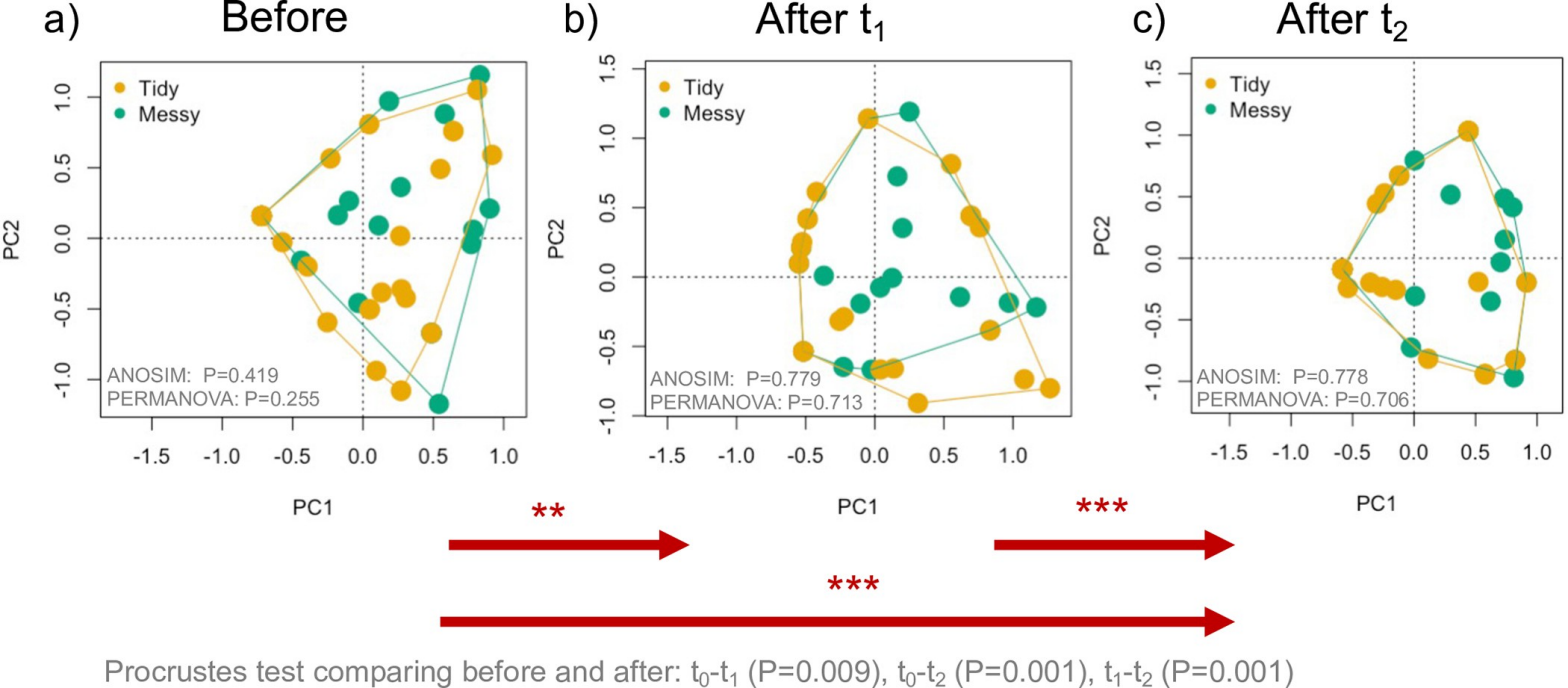
Changes in arthropod natural enemy community composition through the duration of the experiment at the order taxonomic level. Each point on the plot represents the composition of individuals within orders found within pitfall traps and visual observations within a garden bed, within a given treatment (tidy, messy). Singleton species were removed from this analysis. Significant differences in community composition between treatments were tested with ANOSIM and PERMANOVA, and differences between the time points (t_0_ and t_1_, t_1_ and t_2_, t_0_ and t_2_) were tested using Procrustes tests. Arrows and asterisk indicate significant differences between natural enemy communities within garden beds between time points for the Procrustes tests (Significance: ‘*’ 0.05, ‘**’ 0.01, ‘***’ 0.001).

In testing how individual garden beds differed or changed between time points, the tidy/messy manipulation significantly shifted community composition of natural enemies within individual garden beds, with communities significantly different from one another after the treatment (Fig 3). The Procrustes test found that immediately after the experiment (t_1_) composition within each garden bed was significantly different from that same bed sampled before the manipulation (Procrustes test t_0_–t_1_: Procrustes Sum of Squares (m12) = 0.837; Correlation term = 0.404; Significance = P = 0.009), and also from t_2_ (t_1_–t_2_: m12 squared = 0.785; Correlation term = 0.463; P = 0.001). We found differences between communities within garden beds before the manipulation and t_2_ (t_0_–t_2_: m12 squared = 0.809; Correlation term = 0.437; P = 0.001).

### Effects on predation services

The tidy/messy manipulation influenced predation services, but had different effects on removal of the two pest species (Table 2 and Fig 2). For aphids, predation differed between treatments immediately after the manipulation at t_1_ (Fig 2), with higher aphid removal in the tidy treatment and lower aphid removal in the messy treatment (Wilcoxon rank sum test; P = 0.02), but at t_2_, predation shifted towards similar pre-manipulation predation service levels in both treatments. No factors strongly predicted post-manipulation aphid removal, while the best model predicting egg removal included treatment, VCI and mulch present in the garden (Table 2). Egg removal was significantly lower in gardens with more mulch ground cover (P = 0.02; Table 2). The tidy/messy manipulation in relation to egg predation showed a decrease in egg removal over time in the messy treatment than in the ‘tidy’ treatment (Fig 2).

## Discussion

Management decisions in urban agroecosystems are determined by human preferences and values to thereby influence biodiversity and species interactions that underpin ecosystem services [45, 46]. Yet aesthetic preferences towards orderly, tidy ecosystems that direct management decisions can create tensions with desires for the ecological function of the system [7]. We explore this tension by testing how a popular “tidy” management practice in urban gardens may influence predation services. Within the time span of our experiment, we found that this may have some short-term changes in some predation services, but may not provide long-term benefits. Yet, changes in biodiversity or service provision may depend on the time since the management implementation, and what management features are already present in the system (e.g., vegetation cover and diversity, ground cover). We discuss the two main findings of our experiment: *(i)* that altering tidiness or messiness can modulate natural enemy communities within garden beds in the short-term; and *(ii)* that increasing tidiness does not drastically reduce or sustain long-term pest control benefits. We conclude with recommendations for future research to investigate long-term effects as well as recommendations for urban gardeners.

### Influence of garden tidiness on natural enemy communities

Our first finding is that altering the ‘tidy’ or ‘messiness’ of a garden can influence the assemblage of arthropod natural enemies within garden beds within a short time period. In support of our hypothesis, we found that communities changed within beds after the tidy/messy manipulation, suggesting that changing mulch and weed cover in pathways between garden beds may have influenced natural enemy movement (e.g., through enhanced connectivity from vegetation or increased isolation due to weed removal and mulch addition) and subsequent community assembly within this short time span. Thus, while there were no strong differences between herbivore and natural enemy abundance or richness between tidy and messy treatments, the manipulations did change the community composition within garden beds. This has implications for pest management, as natural enemy composition can be more important than richness for pest suppression due to how the diverse functional roles of natural enemies complement one another within an ecological community [47, 48].

Changes in connectivity between beds through an increase or decrease in vegetation availability may be one potential mechanism driving community (dis)similarity [49], as we hypothesized (*H2*). Connectivity is important in agricultural systems for increasing movement of natural enemies and thereby promoting predation services [50]. Here, management practices that alter agroecosystem connectivity through vegetation management influence natural enemies’ ability to find (crop) pests, or for pests to find their hosts. The enemies hypothesis would predict that adding vegetation directly creates more niches for a greater diversity of natural enemies or indirectly provides more prey resources [1, 51, 52]. If we apply island biogeography theory [53] to the scale of garden beds, one might predict that increasing tidiness creates garden plot ‘islands’ surrounded by a pathway ‘sea’ of mulch groundcover. Such island biogeography patterns have been found in urban and agricultural landscapes [54–56], for example, where weedy strips and vegetated “island” habitats serve as reservoirs for natural enemies within fields (e.g., “Beetle Banks” for Carabids; [57]). In an urban garden system, mulch may produce an impermeable matrix for some organisms preventing dispersal between garden beds but may also promote the activity of other natural enemies. This may thereby facilitate the creation of distinct communities. For example, in our system, activity of ground-dwelling natural enemies such as spiders increases with more mulch [58]. Alternatively, more vegetation connectivity in ‘messy’ areas may promote natural enemy movement to facilitate or maintain community similarity [49, 59], or may reduce intraguild predation by increasing prey or habitat availability [2].

Our work provides some support for the idea that natural enemy assemblage and movement can be sensitive to changing habitat conditions [48, 60, 61]. Whatever the disturbance—in our case, whether adding mulch or adding vegetation to a garden—could immediately influence community assembly and composition within a garden bed. Although the relative contributions of natural enemy groups to treatment dissimilarity was relatively similar across all the time points, the difference in species composition at the bed level is significant. This suggests that management decisions by individual gardeners in and surrounding their garden beds may indeed influence the natural enemies and potentially pest control in their gardens. Moreover, the results also indicate that this microscale of a garden bed or plot within a larger garden may indeed be a relevant scale at which to look at arthropod community change.

### Influence of garden messiness and tidiness on predation services

The tidy/messy manipulation affected initial aphid but not egg pest predation. Interestingly, the change in aphid predation was significantly higher in the tidy area than in the messy area after the manipulation. The change in aphid predation and subsequent differences between manipulation areas could be due to changes in lady beetle and Argentine ant (*Linepithema humile*) activity, as *L*. *humile* presence and abundance correlates with shifts in composition and diversity of other invertebrates (e.g., [62, 63]). In our system, the most likely aphid predators are lady beetles, many of which are aphidophagous [64]. Yet ant activity may reduce effective aphid predation by lady beetles through aphid tending, an interaction in which ants protect aphids from predation as they farm aphids for honeydew. Thus, changes in ant and lady beetle activity in the treatment areas would then likely predict changes in aphid predation services. While common aphidophagous species like *Hippodamia convergens* were consistently observed in both messy and tidy areas at all time points, *L*. *humile* captures were consistent in the messy areas across all time points, while captures in the tidy area strongly dropped (by 50%) at t_1_ then increased to near pre-manipulation numbers at t_2_ (S2 Fig). These changes in ant abundance in manipulation areas could explain the differences in aphid predation between treatments that narrowed over time: consistent ant abundance in the messy area could have potentially affected lady beetle access to aphids, therefore lowering aphid predation there. In our study, we did observe, but did not make careful quantification of ant presence on plants with aphids. It would be interesting to further explore long-term impacts of the treatment on ants and their interactions with other natural enemies to better elucidate the exact mechanism driving changes in predation services in relation to ‘tidiness’.

The manipulation’s lack of a strong consistent effect on predation services over time may be an interesting result of our work. Though our experiment was short term, there may be no downside to having some messiness, nor a large positive effect of adding tidiness for predation services.

### Future research directions

This study provides further understanding of the intersections between aesthetics, biodiversity, and ecosystem service provision in urban agroecosystems. Our study is temporally and spatially limited, but we hope to guide future research. First, our assessment of the manipulation is temporally limited (seven days), and we do not know the long-term impacts of such habitat manipulations. Although arthropods certainly move very quickly and urban gardens are highly dynamic systems, we may not have captured the full extent of the impacts on recolonization dynamics, and we acknowledge that our broad taxonomic identification of natural enemies limits our understanding of how species identity and the assemblage of communities is influenced by this practice and thereby predation services. Furthermore, an interesting question is how the ‘tidy/messy’ structural changes could affect the communities of natural enemies and herbivores in the following seasons. Future work should further explore how community composition changes over time and work at finer taxonomic levels. Second, we used the mulch woodchip manipulation as a representation of a ‘tidy’ agroecosystem management practice. Yet, not just the physical properties of the woodchips but its chemical properties are important considerations for future research into ecological mechanisms driving changes in biodiversity and ecosystem function. For example, many types of mulch have chemical properties that could impact natural enemy behavior because many specialist arthropod natural enemies including parasitoid wasps and jumping spiders rely on olfactory cues to locate prey [65]. Third, we did not control for the vegetation already present in the messy treatment, meaning that we cannot disentangle the effects of vegetation we added to the system and the ‘weeds’ already present in the system. Fourth, here we make the assumption that mulching is an aesthetic practice used by the gardeners, but we did not ask the gardeners about their perceptions around mulch in relation to their management decisions. Future ecological experimental work could integrate social research on how gardeners’ perceptions and aesthetic preferences influence their management decisions.

## Conclusion

Tidy versus messy aesthetic-driven management practices can influence changes in arthropod biodiversity and related ecosystem services, but this likely depends on the organism and service of focus. Our tidy/messy manipulation experiment indicates that there are often not linear outcomes of implementing ‘messy’ versus ‘tidy’ gardening practices. Direct management suggestions and ecological application are therefore not straightforward. This is, in and of itself, a barrier to advocating for garden messiness, which is hard to manage and predict, and thus is likely more difficult to advertise to gardeners. However, nor are our results an advertisement for implementing tidy systems. Messiness did not boost pest populations, nor did tidiness provide a big boost to pest control. Thus, small steps can be taken to recognize that aesthetic preferences, biases and management strategies towards simplified ‘tidy’ systems may optimize ecosystem service provision. Rather, we recommend that urban gardeners and farmers can forgive some spontaneous vegetation in their gardens, and recognize that ‘messy’ ecological practices can maintain ecological function, and also show stewardship, not only neglect.

## Supporting information

S1 Fig Set up of the experimental design. The simplified diagram shows the tidy treatment area and the messy treatment area within the 20 m x 20 m sampling area in the gardens. Both treatment areas had four beds that were monitored throughout the experiment, including having a sticky trap and pitfall trap within the bed. Sentinel pest plants in pots were placed within the treatment area along with potted plants to the messy treatment area.
(PDF)

S2 Fig Trends in the changes in Argentine ants (*L*. *humile*) across the experiment. Specifically, changes in raw values after the first and second observations. The graph shows the raw value means across the 16 beds within each treatment area in each of the eight gardens. Positive values indicate a higher response variable value after the experiment, whereas negative values indicate a lower response variable value. Error bars represent standard errors (SE) of the means. Time points include three days after the manipulation (t_1_) and seven days after the manipulation (t_2_).
(PDF)

S1 Data (XLSX)
