# Supplementary material for: ‘Tidy’ and ‘messy’ management alters natural enemy communities and pest control in urban agroecosystems
Source: PLoS One. 2022 Sep 22;17(9):e0274122. doi: 10.1371/journal.pone.0274122 (PMC9499222; doi:10.1371/journal.pone.0274122)
Supplement: S1 Fig — The simplified diagram shows the tidy treatment area and the messy treatment area within the 20 m x 20 m sampling area in the gardens. Both treatment areas had four beds that were monitored throughout the experiment, including having a sticky trap and pitfall trap within the bed. Sentinel pest plants in pots were placed within the treatment area along with potted plants to the messy treatment area. (PDF) [file pone.0274122.s001.pdf]

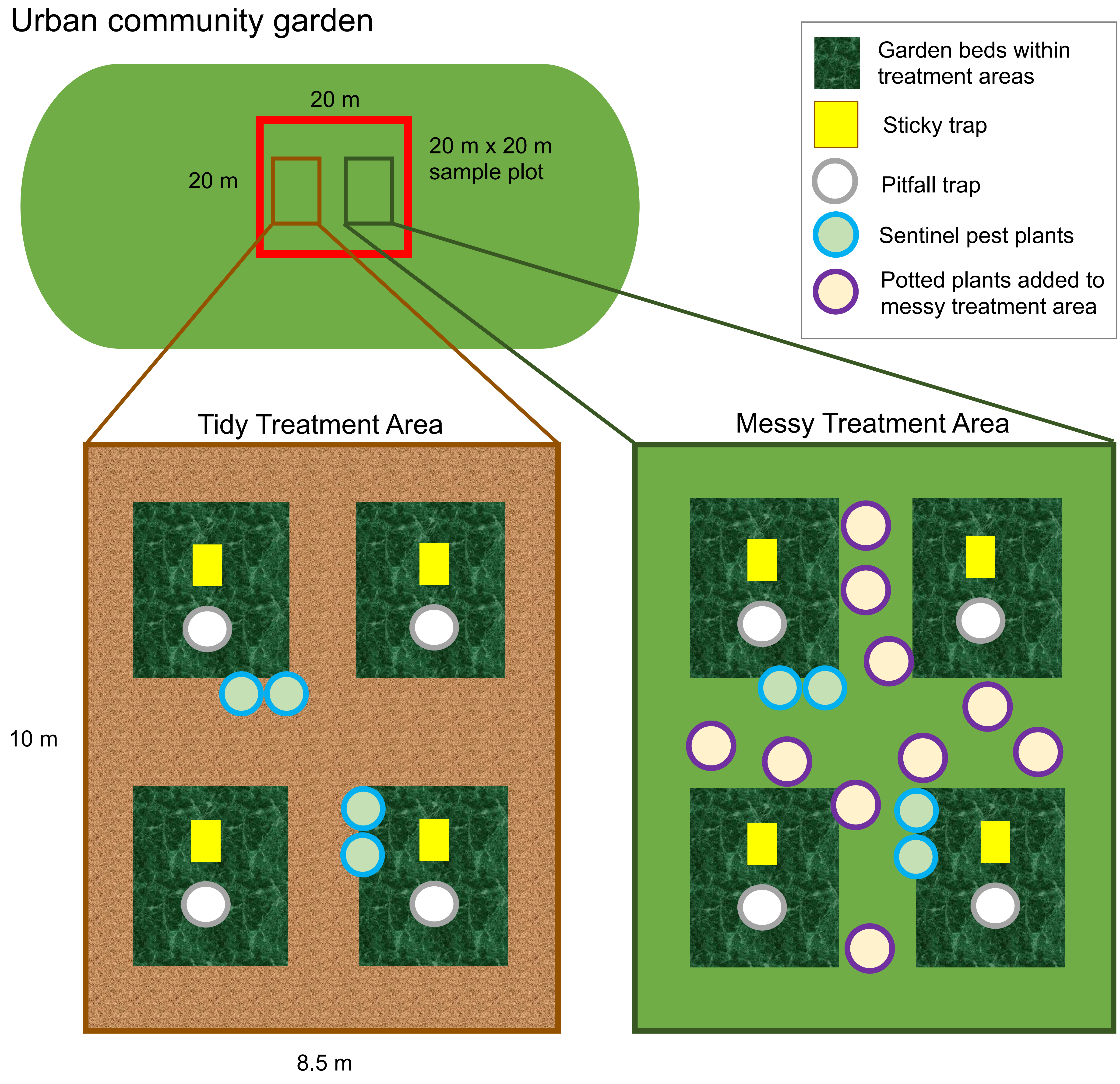
Urban community garden
20 m
20 m
20 m x 20 m sample plot
Garden beds within treatment areas
Sticky trap
Pitfall trap
Sentinel pest plants
Potted plants added to messy treatment area
Tidy Treatment Area
Messy Treatment Area
10 m
8.5 m
