# Supplementary figures and images for: ‘Tidy’ and ‘messy’ management alters natural enemy communities and pest control in urban agroecosystems
Source: PLoS One. 2022 Sep 22;17(9):e0274122. doi: 10.1371/journal.pone.0274122 (PMC9499222; doi:10.1371/journal.pone.0274122)

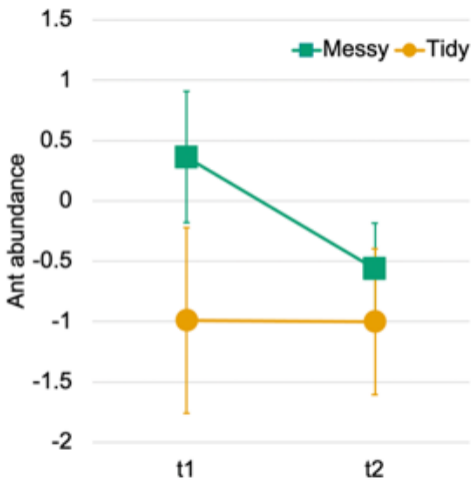

Supplement: S2 Fig — Specifically, changes in raw values after the first and second observations. The graph shows the raw value means across the 16 beds within each treatment area in each of the eight gardens. Positive values indicate a higher response variable value after the experiment, whereas negative values indicate a lower response variable value. Error bars represent standard errors (SE) of the means. Time points include three days after the manipulation (t1) and seven days after the manipulation (t2). (PDF) [file pone.0274122.s002.pdf]
